# Early Adjacent-Level Vertebral Fracture Following Vertebroplasty in Severe Osteoporosis: A Case Report

**DOI:** 10.7759/cureus.104754

**Published:** 2026-03-06

**Authors:** Niraj K Choudhary, Jeevesh Mallik, Manoj Kumar, Dattatraya Mallik

**Affiliations:** 1 Neurosurgery, Tata Main Hospital, Jamshedpur, IND

**Keywords:** adjacent vertebral fracture, lumbar spine, osteoporosis, osteoporotic vertebral fracture, percutaneous vertebroplasty

## Abstract

Osteoporotic vertebral compression fractures are a common cause of back pain and disability in elderly patients. Percutaneous vertebroplasty is frequently performed to achieve pain relief in patients who do not respond to conservative treatment. However, new vertebral fractures, including fractures at adjacent levels, may occur following the procedure. We report the case of an 87-year-old woman with a severe osteoporotic L2 vertebral compression fracture treated with vertebroplasty who developed an adjacent-level L1 fracture within two weeks. The clinical course, imaging findings, and potential contributing factors are described. This case highlights severe osteoporosis as a major risk factor for early adjacent-level vertebral fracture after vertebroplasty.

## Introduction

Osteoporotic vertebral compression fractures are a frequent cause of pain, reduced mobility, and diminished quality of life in elderly patients. The occurrence of a single vertebral fracture substantially increases the risk of subsequent fractures, particularly during the first year following the index fracture [1].

Percutaneous vertebroplasty is commonly used to stabilize painful osteoporotic vertebral fractures and provide rapid pain relief when conservative treatment fails [2]. Although vertebroplasty is effective for symptom control, new vertebral fractures have been reported after the procedure, including fractures at adjacent vertebral levels [3,4]. The etiology of these fractures is considered multifactorial, involving altered spinal biomechanics and underlying bone fragility [5].

Early occurrence of adjacent-level vertebral fracture following vertebroplasty remains clinically important because it may influence patient counselling, prognostication, and post-procedural management strategies. Reporting such cases contributes to a better understanding of potential risk factors and emphasizes the need for comprehensive osteoporosis management following vertebral augmentation.

## Case presentation

An 87-year-old woman presented in October 2025 with sudden-onset low back pain without a history of significant trauma. The pain was mechanical in nature, aggravated by movement, and severely limited ambulation. There were no associated neurological deficits or bowel and bladder disturbances. Her medical history was significant for hypertension, for which she was on regular medication.

**Figure 1:**
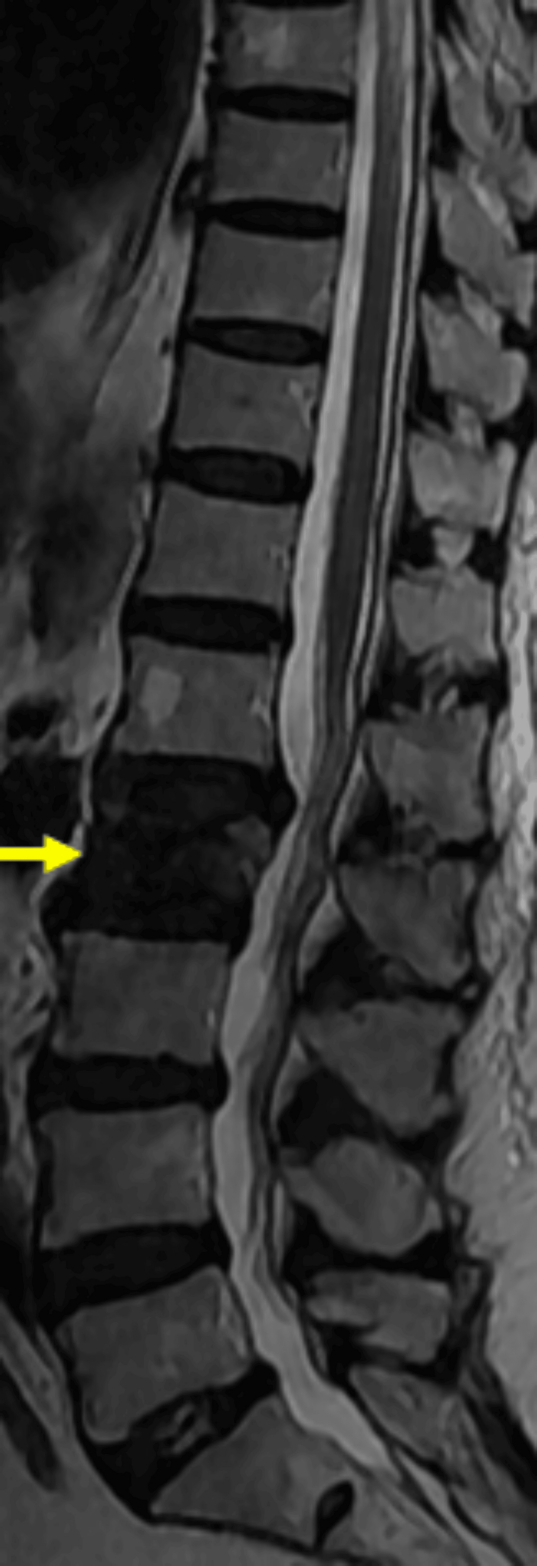
Pre-procedure sagittal T2-weighted magnetic resonance imaging of the thoracolumbar spine demonstrating an acute osteoporotic compression fracture of the L2 vertebral body, characterized by loss of vertebral height and altered marrow signal intensity (yellow arrow).

Dual-energy X-ray absorptiometry revealed severe osteoporosis with a T-score of -4.2. Plain radiographs of the lumbosacral spine demonstrated a compression fracture of the L2 vertebral body. Magnetic resonance imaging demonstrated an altered marrow signal within the L2 vertebral body on a sagittal T2-weighted sequence, consistent with an acute osteoporotic compression fracture. The adjacent vertebral bodies were preserved, and there was no evidence of posterior wall breach or spinal canal compromise (Figure 1).

Computed tomography of the chest revealed patchy ground-glass opacities, and computed tomography of the abdomen showed multiple simple hepatic cysts. These findings were considered incidental and unrelated to the spinal pathology. Initial conservative management consisted of oral paracetamol 650 mg three times daily, intermittent nonsteroidal anti-inflammatory medication as required, and thoracolumbar bracing. Despite approximately two weeks of conservative therapy, the patient continued to experience severe pain (visual analog scale score 8/10) with significant limitation in mobility. Percutaneous vertebroplasty was therefore performed at the L2 level under fluoroscopic guidance using a transpedicular approach. The procedure was uncomplicated, and the patient experienced significant pain relief within 24 hours.

**Figure 2:**
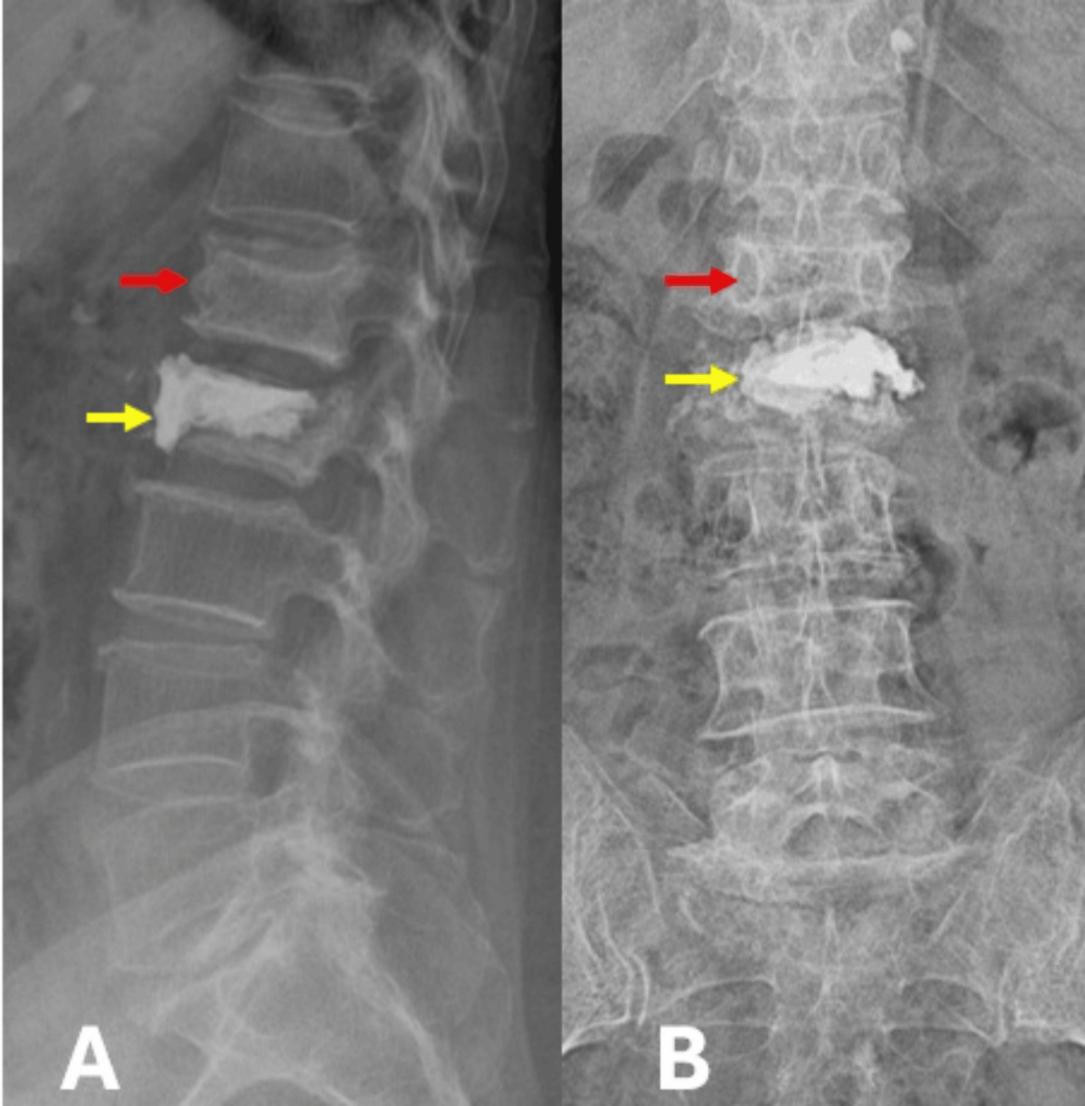
(A) Post-vertebroplasty lateral-view plain radiograph of the lumbar spine showing polymethylmethacrylate cement within the L2 vertebral body (yellow arrow) and a new wedge compression fracture of the adjacent L1 vertebra (red arrow). (B) Anteroposterior view confirming cement deposition in the L2 vertebral body (yellow arrow) with adjacent-level vertebral collapse at L1 (red arrow).

Approximately two weeks after the procedure, the patient developed recurrent low back pain localized above the treated level, without any new traumatic event. Neurological examination remained normal. A follow-up plain radiograph of the lumbosacral spine, including lateral and anteroposterior views, demonstrated polymethylmethacrylate cement in situ within the L2 vertebral body, along with a new wedge compression fracture of the adjacent L1 vertebra (Figures 2A, 2B).

**Figure 3:**
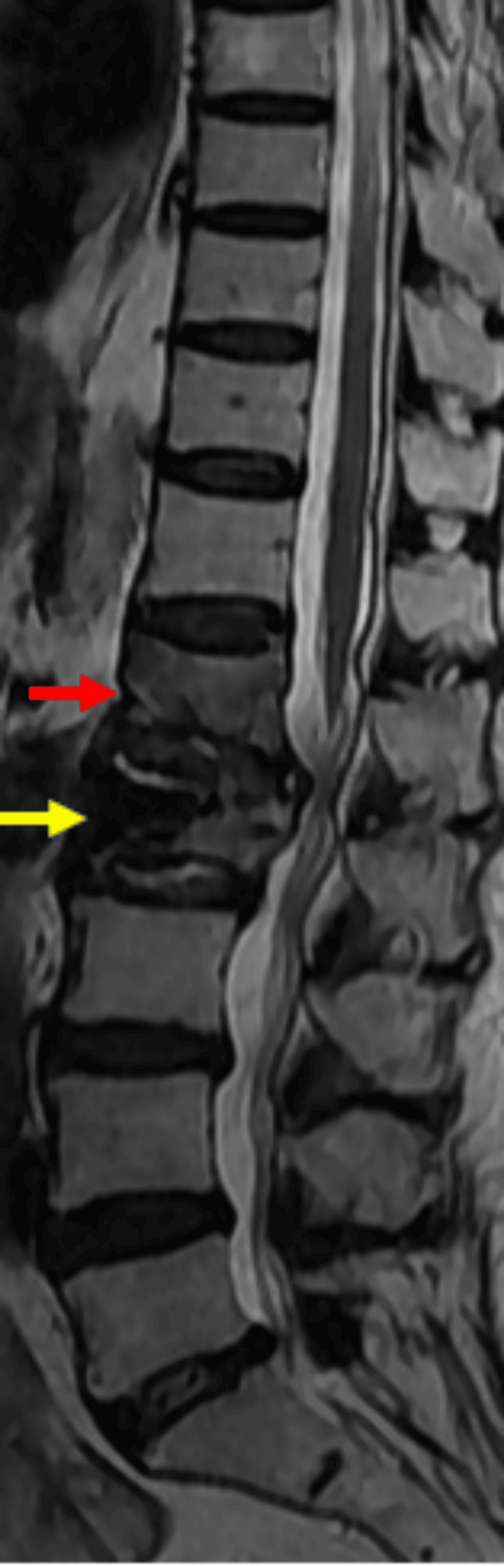
Follow-up sagittal T2-weighted magnetic resonance imaging of the thoracolumbar spine showing post-vertebroplasty status at L2 (yellow arrow) with a new adjacent-level compression fracture involving the L1 vertebral body (red arrow).

Follow-up sagittal T2-weighted MRI demonstrated altered marrow signal with associated vertebral body height loss at the adjacent L1 level, consistent with a new acute compression fracture, while the cemented L2 vertebra remained structurally intact (Figure 3).

The patient was managed conservatively with oral analgesics, thoracolumbar bracing, and initiation of anti-osteoporotic therapy consisting of calcium and vitamin D supplementation along with antiresorptive treatment. At the three-month follow-up, the patient reported a significant reduction in pain (visual analog scale score 2/10) and improved ambulation with minimal assistance. No additional vertebral fractures were identified during follow-up.

## Discussion

Adjacent-level vertebral fractures following vertebroplasty have been reported with variable incidence in the literature [3,4]. Cement augmentation increases the stiffness of the treated vertebra, which may alter load transmission and increase mechanical stress on adjacent osteoporotic vertebrae [3]. Cement leakage into the intervertebral disc has also been associated with an increased risk of adjacent vertebral fractures due to its effect on disc biomechanics [4].

Patient-related factors play a significant role in the development of new vertebral fractures. Advanced age, severe osteoporosis, and low bone mineral density are strong predictors of subsequent fractures [1,5]. In the present case, the markedly reduced bone mineral density (T-score -4.2) and advanced age likely contributed to the early adjacent-level fracture rather than technical failure of the vertebroplasty procedure.

Several studies have demonstrated that although vertebroplasty provides effective short-term pain relief, it does not significantly reduce the overall risk of future vertebral fractures compared with conservative treatment [2,6]. Vertebroplasty should therefore be regarded as a symptomatic treatment, and comprehensive management of osteoporosis is essential to reduce the risk of subsequent fractures.

Importantly, adjacent-level fractures following vertebral augmentation are not considered exclusive complications of vertebroplasty. Several comparative studies have reported similar rates of subsequent vertebral fractures following vertebroplasty, kyphoplasty, and even conservative management, indicating that underlying osteoporosis and intrinsic skeletal fragility remain major determinants of fracture progression rather than procedural biomechanics alone [7,8]. Risk factor analyses have consistently demonstrated that advanced age, low bone mineral density, and prior vertebral fractures significantly increase the likelihood of new vertebral fractures after vertebral augmentation procedures [9]. Furthermore, clinical studies evaluating outcomes after vertebroplasty have shown that while the procedure provides rapid pain relief and improved mobility, it does not substantially modify the natural history of osteoporotic fracture risk [10]. These findings support the interpretation that the early adjacent-level fracture observed in our patient most likely reflects severe underlying osteoporosis combined with altered segmental biomechanics rather than an isolated technical complication.

## Conclusions

Percutaneous vertebroplasty can provide effective pain relief in elderly patients with osteoporotic vertebral compression fractures; however, early adjacent-level fractures remain a recognized complication, particularly in the presence of severe osteoporosis. This case highlights that new vertebral fractures may occur within a short interval following vertebral augmentation, even when the treated level is technically satisfactory. Meticulous patient selection, early initiation of comprehensive osteoporosis management, and close post-procedure follow-up are essential to reduce the risk of subsequent fractures and to optimize long-term outcomes.
